# PUDU (pipeline for universal diversity unveiling): an accessible end-to-end workflow for taxonomic profiling and ecological visualization of environmental microbiomes across amplicon, shotgun, and long-read sequencing

**DOI:** 10.3389/fbinf.2026.1909327

**Published:** 2026-08-11

**Authors:** Alejandro Medaglia-Mata, Pablo Rojas-Rodríguez, Vojtěch Bystrý, Rossy Guillén-Watson, Olman Gómez-Espinoza, Kattia Núñez-Montero

**Affiliations:** 1 Central European Institute of Technology, Masaryk University, Brno, Czechia; 2 Department of Biology, Faculty of Medicine, Masaryk University, Brno, Czechia; 3 University of Potsdam, Potsdam, Brandenburg, Germany; 4 Facultad de Ingeniería, Instituto de Ciencias Aplicadas, Centro de Investigación e Innovación, Universidad Autónoma de Chile, Huechuraba, Chile; 5 Facultad de Ciencias de La Salud, Instituto de Ciencias Aplicadas, Centro de Investigación e Innovación, Universidad Autónoma de Chile, Huechuraba, Chile

**Keywords:** 16S rRNA, bracken, dada2, EMU, kraken, long-read sequencing, metagenomics pipeline, metataxonomics

## Abstract

**Background:**

Environmental microbiome research has advanced through three complementary sequencing modalities, targeted 16S rRNA amplicon sequencing, whole-genome shotgun (WGS) metagenomics, and long-read full-length 16S rRNA profiling, each supported by distinct toolsets with heterogeneous outputs, variable configurations, and different levels of reproducibility documentation. Existing pipelines are typically modality-specific, require substantial configuration expertise, or produce outputs that need further custom scripting before standard ecological analyses can begin. This analytical fragmentation introduces avoidable technical variability and complicates cross-study reproducibility and comparability. PUDU addresses this by integrating all three modalities into a single reproducible workflow with simplified configuration, harmonized outputs across classifiers, and direct compatibility with downstream ecological analysis frameworks.

**Results:**

We present PUDU (Pipeline for Universal Diversity Unveiling), a modular Snakemake workflow that supports amplicon (short-read 16S), shotgun metagenomics (WGS), and long-read 16S analyses from raw reads to standardized outputs for downstream microbial ecology. PUDU performs technology-aware preprocessing and centralized quality control, and integrates established taxonomic approaches, including DADA2 for amplicons, Emu for full-length 16S long reads, and Kraken2/Bracken and Centrifuger for WGS. Across methods, PUDU produces harmonized count and relative-abundance tables at user-defined taxonomic ranks, Krona files, and a standardized Phyloseq-compatible R object to streamline diversity analyses and statistical workflows. PUDU also provides an integrated Shiny interface for metadata-aware alpha/beta diversity, ordination, community composition, and shared-taxa exploration with exportable figures and taxa tables. We demonstrate PUDU on two publicly available environmental datasets spanning rhizosphere WGS and long-read marine sediment 16S, yielding broadly consistent community-level patterns across classifiers (Spearman ρ = 0.936 at phylum level; PERMANOVA R^2^ = 0.87–0.95) with peak memory below 45 GB on a standard Linux workstation.

**Conclusion:**

PUDU is an end-to-end, reproducible, and extensible framework that enables standardized taxonomic profiling and ecology-oriented analysis across sequencing modalities. By combining harmonized outputs, Phyloseq interoperability, and an integrated visualization layer, PUDU facilitates reproducible, standardized, and comparable environmental microbiome analysis from raw reads to interpretable ecological insights.

## Introduction

Microorganisms are fundamental drivers of biogeochemical processes across terrestrial and aquatic ecosystems, regulating nutrient cycling, organic matter decomposition, and interactions between soils, sediments, water bodies, and plant roots (Gibbons and Gilbert, 2015; Lipson and Xu, 2019). Characterizing microbial community structure across environmental gradients is therefore a central objective in microbial ecology, and culture-independent high-throughput sequencing has made this possible at unprecedented scale and resolution (Demko et al., 2021; McIlwaine et al., 2024).

Two strategies dominate current environmental microbiome practice: targeted sequencing of the 16S rRNA marker gene for taxonomic community profiling, and whole-genome shotgun (WGS) sequencing for broader phylogenetic and functional resolution (Shamim et al., 2019; Navgire et al., 2022). More recently, long-read sequencing (Oxford Nanopore and PacBio) has expanded the analytical toolkit by enabling full-length 16S rRNA gene recovery, improving taxonomic resolution to near-species level under favorable reference database coverage (Sevim et al., 2019). Together, these technologies have brought microbiome profiling within reach of research groups across a wide range of institutional and geographic contexts.

However, expanding access to sequencing technology does not automatically translate into the capacity to analyze the resulting data. A particularly important gap exists in research settings where sequencing infrastructure is available, but dedicated bioinformatics expertise is not, a challenge relevant in contexts where sequencing access has outpaced bioinformatics training infrastructure (Krampis, 2022).

Beyond the access limitation, analytical fragmentation is a recognized methodological problem in environmental microbiome research more broadly (Attwood et al., 2019; Rodríguez-Pérez et al., 2022). Workflows that are modality-specific, heterogeneously configured, and non-standardized in their outputs introduce avoidable technical variability that complicates cross-study synthesis and reproducibility (Murigneux et al., 2021; Li et al., 2023). The challenge is amplified by the rapid evolution of long-read sequencing models, the computational burden of large reference databases, and the practical difficulty of keeping heterogeneous tool installations synchronized across computing environments (Attwood et al., 2019; Curry et al., 2022; Dubois et al., 2024).

In this context, we present PUDU (Pipeline for Universal Diversity Unveiling), a modular and reproducible Snakemake-based workflow designed to provide end-to-end environmental microbiome analysis across short-read 16S rRNA gene amplicons, WGS metagenomics, and long-read full-length 16S rRNA gene data. PUDU does not introduce new computational methods for taxonomic classification; instead, it integrates established, field-standard tools such as DADA2 (Callahan et al., 2016) for amplicon ASV inference, Emu (Curry et al., 2022) for long-read 16S rRNA gene marker classification, Kraken2/Bracken (Lu et al., 2017; Wood et al., 2019), and Centrifuger (Song and Langmead, 2024) for WGS profiling, within a reproducible, low-configuration framework that produces harmonized, outputs directly compatible with Phyloseq-based ecological workflows. All workflow parameters are centralized in a single YAML configuration file, all software dependencies are managed via Conda, and outputs are formatted for direct compatibility with the Phyloseq R ecosystem (McMurdie and Holmes, 2013) and an integrated Shiny visualization interface (PUDUVisR). The goal of PUDU is to provide a reproducible, standardized analytical framework that enables environmental scientists with basic command-line familiarity to move from raw sequencing reads to interpretable community-level ecological insights, ensuring consistent, harmonized outputs and full analytical provenance across studies and sequencing modalities.

## Methods

### Software architecture and workflow design

The PUDU pipeline is implemented as a modular workflow using Snakemake (v7.0 or higher), a rule-based workflow management system designed to ensure scalability, parallel execution, and reproducibility. All software dependencies are encapsulated in per-rule Conda environments, enabling portable and reproducible execution on both local workstations and high-performance computing (HPC) systems without conflicts between tool versions. Snakemake was selected over alternative workflow managers such as Nextflow due to its native Python-based rule syntax familiar to the bioinformatics community and reduced configuration overhead for workstation deployment, supporting the accessibility objectives of PUDU.

**FIGURE 1:**
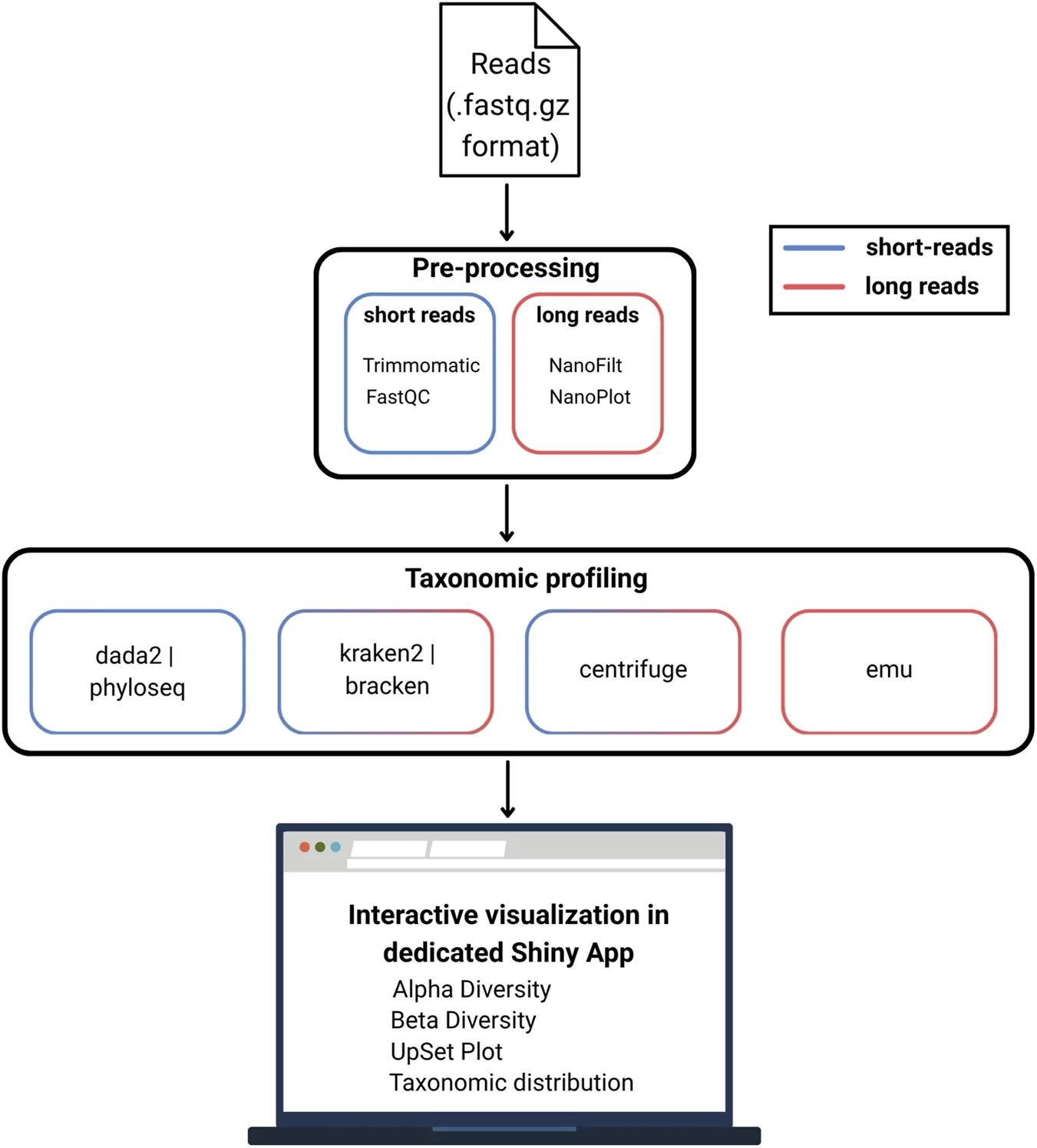
Workflow design of PUDU. Illustrating the workflow structure under three main categories: preprocessing for quality control, taxonomic profiling and interactive visualization.

The workflow is organized into three operational stages: preprocessing and quality control, taxonomic profiling, and output standardization. Users select which modules to activate via the central YAML configuration file (config.yaml); a template is provided with the repository (Supplementary Data 1). All sample identifiers, database paths, filtering thresholds, and module selections are defined in this single file, so that no workflow code requires modification between datasets. Figure 1 summarizes the overall workflow structure.

### Installation and database setup

PUDU requires Conda (Miniconda or Anaconda) and Snakemake. Conda was selected over container-based solutions (e.g., Docker, Singularity) because it requires no administrative privileges, integrates natively with Snakemake--use-conda flag, and is the dependency management approach most familiar to the target user community. PUDU targets environmental scientists with basic command-line familiarity and basic experience with Conda environment management, sufficient to install dependencies, configure a YAML file, and execute a single Snakemake command, without requiring prior experience with workflow management systems or custom bioinformatics scripting. Installation and execution require three steps: cloning the repository, editing the config. yaml with sample names, database paths, and module selection flags, and running a single Snakemake command.

Reference databases must be obtained separately and their paths specified in config. yaml. In this study, the following databases were used: Kraken2 RefSeq complete bacterial, archaeal, and viral genomes (downloaded 30 October 2025); Centrifuger GTDB r226 pre-built index (downloaded 30 October 2025); Emu RDP database version 11.5 (downloaded 30 October 2025); DADA2 SILVA release 138.1 (recommended for amplicon studies). Peak RAM usage under the 10-thread workstation configuration used in this study is reported in Supplementary Table S1; users with less than 32 GB RAM should consider using the Centrifuger GTDB reduced index or the Kraken2 standard-8 database.

### Preprocessing for quality control

Preprocessing is adapted to the sequencing technology. Short-read datasets are processed using Trimmomatic (Bolger et al., 2014), performing adapter removal, quality trimming, and length filtering. Meanwhile, long-read datasets are processed with NanoFilt (De Coster et al., 2018) to apply quality and length cutoffs. Quality control is systematically performed on both raw and filtered datasets using FastQC (Babraham bioinformatics, 2027) for short-reads and NanoPlot (De Coster et al., 2018) for long-reads. All QC outputs are aggregated in MultiQC (Ewels et al., 2016), generating interactive summary reports that enable comparative assessment across samples and runs. This centralized QC facilitates the identification of quality issues and batch-level variation prior to downstream analysis.

### Taxonomic profiling

PUDU supports four taxonomic classification strategies, selectable and combinable via the configuration file:

#### DADA2 (amplicon short-read 16S rRNA gene)

Performs error-model-based amplicon sequence variant (ASV) inference, including quality filtering, dereplication, denoising, chimera removal, and taxonomic assignment against a user-specified reference database (SILVA 138.1 recommended). DADA2 was selected as the amplicon processing module given its validated performance in ASV inference and superior taxonomic resolution relative to OTU-based approaches (Callahan et al., 2016). Output is a Phyloseq-compatible RDS object containing the ASV table, assigned taxonomy, and sample metadata.

#### Emu (long-read full-length 16S rRNA gene)

Performs species-level taxonomic classification of Oxford Nanopore full-length 16S rRNA gene amplicons using an expectation-maximization algorithm optimized for long-read error profiles. Emu was selected as it was designed and validated for ONT 16S reads (Curry et al., 2022). Output includes per-sample relative abundance tables and is exportable as a Phyloseq-compatible RDS object.

#### Kraken2/bracken (16S rRNA targeted or WGS)

Rapid k-mer exact-matching classification against a user-defined reference database, with Bracken re-estimation of relative abundances at user-selected taxonomic ranks (Lu et al., 2017; Wood et al., 2019). In this study, the RefSeq complete bacterial, archaeal, and viral genome database was used.

#### Centrifuger (16S rRNA targeted or WGS)

An alternative classifier optimized for large-scale metagenomics datasets via lossless compression of the reference index (Song and Langmead, 2024). Centrifuger output is processed through a Bracken-compatible re-estimation step and converted into Kraken-style report, enabling consistent downstream handling with Kraken2 results. In this study, the GTDB r226 pre-built index was used.

### Standardized outputs and data structures

All modules produce outputs following a consistent directory structure and naming convention (Table 1). In addition to module-specific classification reports, PUDU generates: (i) relative abundance tables at user-specified taxonomic ranks; (ii) Krona-compatible files (Ondov et al., 2011) for hierarchical interactive visualization; and (iii) Phyloseq-compatible RDS objects for downstream diversity and statistical analysis in R. This standardized output contract is the feature that primarily distinguishes PUDU from running its constituent tools independently: a user receives consistently structured files from all activated modules without post-processing or format conversion.

**TABLE 1:** Description of the outputs generated by PUDU Pipeline for taxonomic profiling.

Step/Tool	Output file	Format	Description
Kraken2	Results/kraken/{sample}_kraken2.txt	TXT	Raw classification report with read counts and taxonomic IDs
Bracken (on Kraken2)	Results/kraken/{sample}_kraken2_report_bracken.txt	TXT	Re-estimated abundances at selected taxonomic levels
Centrifuger	Results/centrifuger/{sample}_centrifuger.tsv	TSV	Classification table with taxonomic IDs and assignment scores
Bracken (on centrifuger)	Results/centrifuger/{sample}_kraken2_report_bracken.txt	TXT	Bracken re-estimation using kraken-style report derived from centrifuger output
Emu	Results/emu/{sample}_rel-abundance.tsv	TSV	Relative abundance table with normalized taxonomic profiles
DADA2	Results/dada2/phyloseq.rds	RDS	Phyloseq object with ASVs, taxonomy, and metadata

### Configuration and reproducibility

All analytical parameters, sample identifiers, filtering thresholds, database paths, taxonomic ranks, and module selection flags are defined in a single YAML configuration file (Supplementary Data 1). This design means that a complete re-analysis of any dataset requires only the config. yaml, the input reads, and the PUDU repository: no custom scripts, no post-hoc file renaming, and no undocumented intermediate steps. Snakemake automatically records software versions and executed commands in log files organized by sample and rule, supporting transparent provenance tracking and facilitating troubleshooting when errors occur. The complete set of parameters used in this study, including all default values and any modified settings are documented in Supplementary Data 1 (config.yaml) and Supplementary Table S2.

### Integrated visualization platform (PUDUVisR)

**FIGURE 2:**
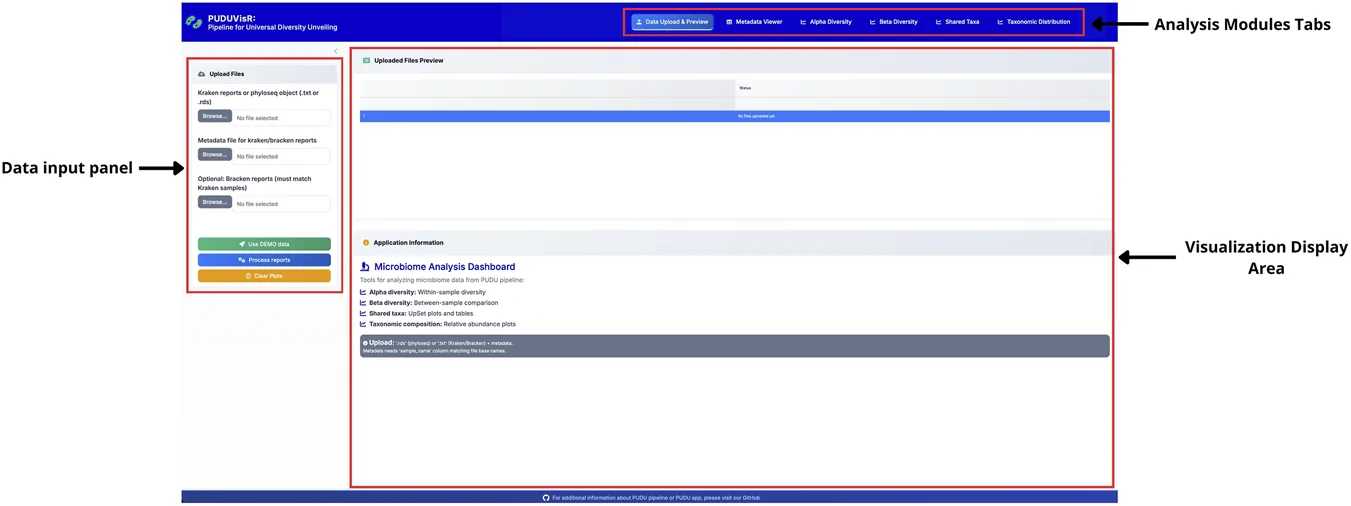
PUDUVisR interactive environment for taxonomic profiling and visualization.

PUDUVisR is a Shiny-based interactive application included with PUDU that operates directly on the standardized pipeline outputs, requiring no additional data preparation. It accepts Kraken2/Bracken and Centrifuger abundance tables and DADA2-derived Phyloseq objects, and provides the following analytical modules: (i) alpha diversity, Shannon, Simpson, and Inverse Simpson indices, displayed as scatterplots, boxplots, or violin plots, with metadata-aware group stratification; (ii) beta diversity, Bray-Curtis dissimilarity-based PCoA, NMDS, and PCA ordinations, with metadata filtering; (iii) taxonomic composition, interactive stacked barplots at user-selected ranks, filterable by sample or metadata variables; (iv) shared-taxa analysis, UpSet-style visualizations for cross-sample or cross-group taxon overlap. All plots and associated taxa tables can be exported in publication-ready formats. PUDUVisR runs in standard web browsers and can be installed from GitHub using devtools::install_github(“amedagliamata/PUDUVisR”). Figure 2 shows the main PUDUVisR interface.

## Results

### Standardized outputs for ecology-oriented analyses

The standardized output structure implemented in PUDU enables direct integration of taxonomic profiles with ecological analysis frameworks, facilitating reproducible comparison of microbial communities across environmental datasets. By maintaining consistent file naming conventions and output formats, the pipeline minimizes post-processing steps typically required to merge classifier results, thus establishing a direct interface between taxonomic assignment and downstream ecological analysis. Table 1 summarizes the main outputs generated by PUDU at each analytical stage.

### PUDUVisR: interactive exploration and export of standardized pipeline outputs

To illustrate the visualization layer integrated with PUDU, Figure 2 shows the main interface of the PUDUVisR Shiny application. The dashboard is organized into modular panels supporting core microbiome ecology tasks, including: (i) alpha diversity metrics (Shannon, Simpson, and Inverse Simpson) presented as scatterplots, boxplots, and violin plots; (ii) beta diversity analyses based on Bray–Curtis dissimilarities with ordination methods such as PCoA, NMDS, and PCA; (iii) UpSet-style visualizations for shared-taxa inspection; and (iv) interactive stacked barplots to summarize taxonomic composition. All modules are metadata-aware, enabling stratification and filtering by covariates. Plots and associated taxa tables can be exported in publication-ready formats, facilitating downstream interpretation while preserving the reproducibility of the underlying command-line workflow.

### Demonstration of workflow functionality and performance on environmental datasets

We evaluated PUDU pipeline on two publicly available datasets representing complementary sequencing modalities: short-read whole-genome shotgun (WGS) sequencing from Antarctic plant rhizosphere samples [BioProject PRJNA419970 (Molina-Montenegro et al., 2019)] and long-read 16S-targeted (v3-v9) sequencing from marine sediments [BioProject PRJNA1153974 (Philip et al., 2025)]. These datasets were selected to illustrate PUDU multi-modal functionality across distinct sequencing technologies and community types; they are not intended to be representative of the full complexity or diversity of environmental microbiome datasets.

### Case study 1: short-read whole-genome shotgun sequencing

#### Dataset and context

To demonstrate PUDU WGS module under real-world conditions, we applied it to a publicly available dataset of whole-genome shotgun metagenomics from Antarctic plant rhizosphere samples (BioProject PRJNA419970). The dataset consists of six samples (three treated, three control) from the rhizosphere of *Deschampsia antarctica*, a vascular plant native to the Antarctic Peninsula.

#### Taxonomic profiling and cross-classifier concordance

For the WGS short-read dataset, PUDU executed its short-read module integrating Kraken2, Centrifuger, and Bracken to generate genus-level taxonomic profiles for each sample. The resulting outputs revealed a consistent community structure dominated by *Streptomyces*, *Pseudomonas*, *Burkholderia*, and *Bradyrhizobium* taxa, consistent with previous reports describing these genera as major rhizosphere constituents under Antarctic and temperate soil conditions.

**FIGURE 3:**
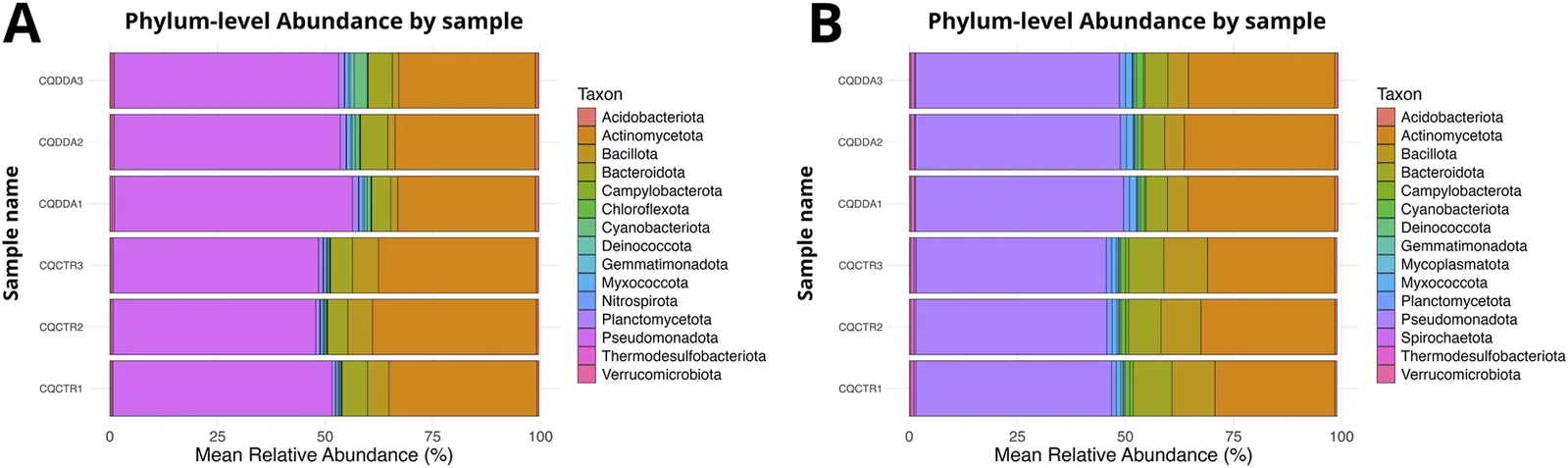
Taxonomic distribution of classified short reads at the Phylum level. **(A)** *Kraken2 classifier.* **(B)** *Centrifuger classifier.* Corresponding taxonomic assignments with differences in genus abundance and unclassified read proportion compared to Kraken2.

To complement these results, a taxonomic distribution plot at the Phylum level was generated, showing highly similar assignment patterns across classifiers, with minor variations in relative abundances (Figure 3).

**FIGURE 4:**
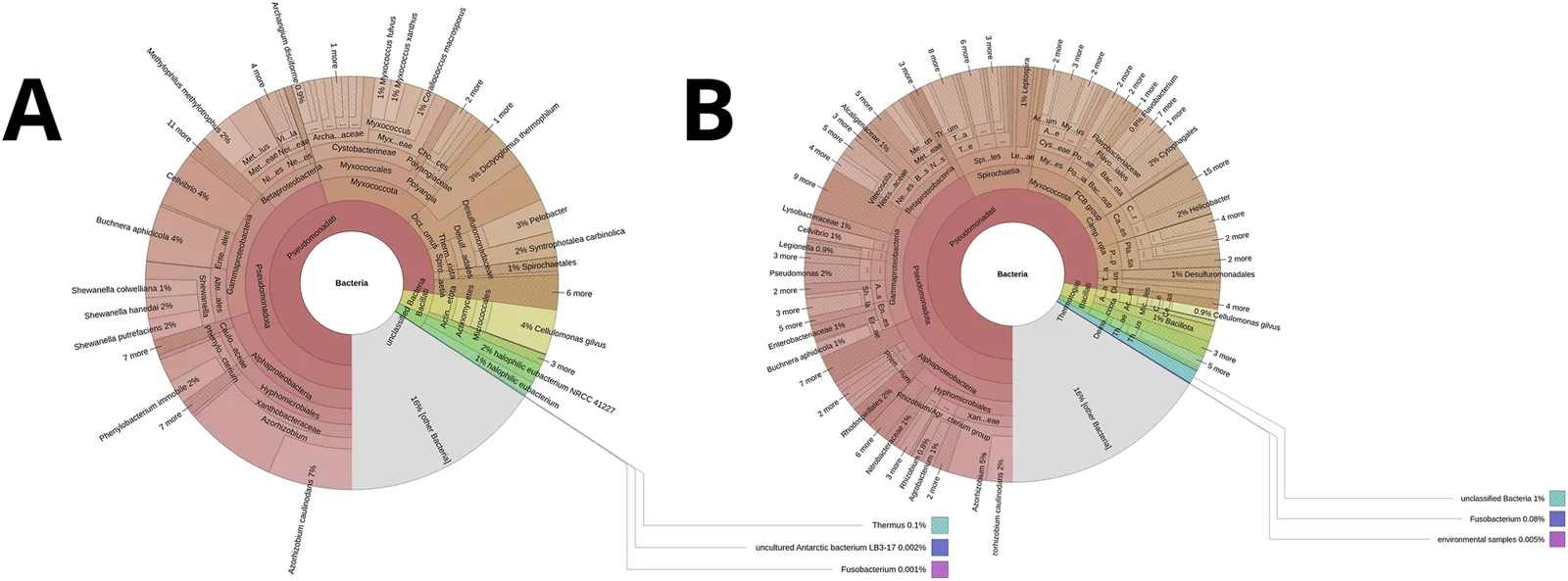
Interactive Krona representation of taxonomic assignments showing hierarchical abundance at multiple taxonomic levels. **(A)** *Kraken2 classifier.* **(B)** *Centrifuger classifier.* Corresponding Krona plot revealing subtle differences in species-level assignments and unclassified read fractions.

The overall bacterial community structure was consistent with the patterns reported in the original publication (Molina-Montenegro et al., 2019), indicating that PUDU standardized processing pipeline preserves community-level taxonomic patterns under the conditions evaluated in this case. These differences are inherent to each classification tool, reflecting the underlying algorithms, reference databases, and taxonomic resolution strategies used. In addition, interactive Krona visualizations were produced for representative samples, allowing direct comparison between Kraken2 and Centrifuger assignments (Figure 4). Both classifiers displayed overlapping taxonomic hierarchies, with subtle differences in the detection of low-abundance families, further illustrating the internal consistency and complementary strengths of the integrated approach implemented in PUDU.

**FIGURE 5:**
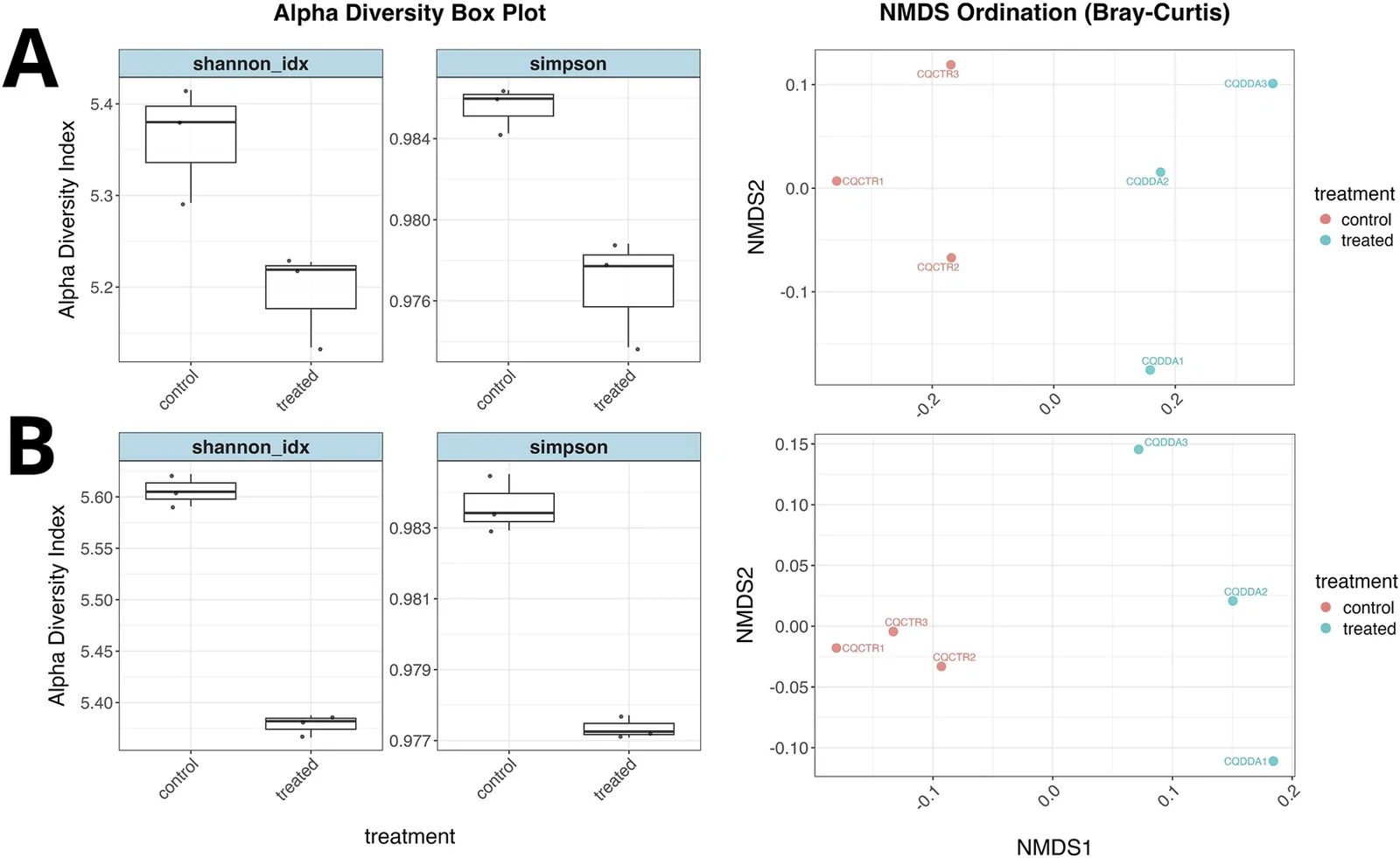
Short-read dataset alpha and beta diversity tool summary. **(A)** Alpha and beta diversity profiles derived from Kraken2 taxonomic assignments using default filtering parameters. **(B)** Corresponding profiles generated with Centrifuger, showing comparable overall diversity patterns but differences in taxonomic resolution at lower ranks.

To further assess the consistency of community structure between classification tools, alpha and beta diversity metrics were compared across Kraken2 and Centrifuger outputs processed through PUDU. Both classifiers exhibited comparable diversity patterns; alpha diversity (Shannon and Simpson indices) showed similar trends between control and treated samples, with only minor variations in magnitude. Likewise, NMDS ordinations based on Bray-Curtis distances revealed consistent clustering of control and treated groups across classifiers; the results are summarized in Figure 5. Kraken2 and Centrifuger recovered consistent community-level patterns when processed under harmonized PUDU outputs. Moreover, the equivalence of diversity metrics confirms that PUDU standardized output structure allows robust downstream analysis independent of the classification algorithm chosen. This stability is critical in ecological microbiome studies, where reproducibility across tools is often limited.

Classifier concordance was quantified at Phylum level using Spearman correlation between abundance profiles derived from Kraken2 and Centrifuger. Across samples, correlation values indicated strong concordance between classifiers (median ρ = 0.936; range = 0.922–0.950), confirming preservation of community-level taxonomic structure despite methodological differences.

#### Diversity patterns and ecological signal stability

Alpha and beta diversity metrics were computed from harmonized PUDU outputs using PUDUVisR (Figure 5). Shannon and Simpson diversity indices showed comparable trends across Kraken2 and Centrifuger outputs, with minor magnitude differences attributable to the known differences in classification stringency between tools. NMDS ordinations based on Bray-Curtis dissimilarities showed consistent grouping of control and treated samples in both classifier outputs. To assess ecological signal preservation, Bray-Curtis dissimilarities were subjected to PERMANOVA analysis; both classifiers yielded high effect sizes (Kraken2/Bracken R^2^ = 0.87; Centrifuger R^2^ = 0.95), indicating structured ecological separation. PERMANOVA did not reach statistical significance (p = 0.1), which is expected given the limited sample size (n = 6 total; 3 per group) and should be interpreted accordingly: these results are presented as a qualitative illustration of PUDU output consistency across classifiers. PERMDISP analysis indicated no significant differences in within-group dispersion (p > 0.3), suggesting that the observed group structure reflects compositional differences rather than variance heterogeneity.

Together, these results demonstrate that PUDU produces consistent, ecologically interpretable outputs from WGS metagenomics data on a standard workstation, and that its harmonized output structure facilitates cross-classifier comparison without additional analytical infrastructure.

### Case study 2: long-read 16S targeted sequencing

#### Dataset and context

To demonstrate PUDU long-read module, we applied it to a publicly available dataset of Oxford Nanopore full-length (V3–V9) 16S rRNA gene sequencing from marine sedijment samples (BioProject PRJNA1153974). This dataset was selected as a complement to the rhizosphere WGS case study: it represents a different sequencing modality and a different environment type. Marine sediment microbiomes are characterized by low biomass and, in chemically stratified environments, can be dominated by a small number of metabolically specialized taxa.

#### Community reconstruction and reproducibility across replicates

**FIGURE 6:**
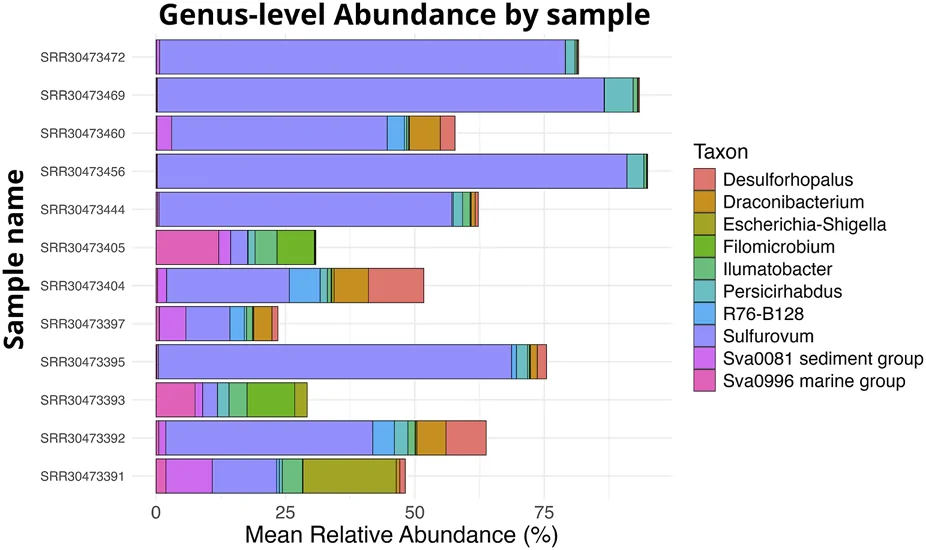
Long-read dataset taxonomy assignment distribution using the Emu classifier. Taxonomic distribution of classified reads at the genus level from long-read sequencing.

PUDU long-read module employed Emu with the RDP 11.5 database for full-length 16S rRNA classification. The analysis reconstructed a community dominated by *Sulfurovum* across all samples, accounting for approximately 85% of relative abundance, with minor taxa each contributing less than 5%. This composition is consistent with expectations for a sulfur-rich marine sediment environment, where *Sulfurovum*, a chemolithotroph, is a documented dominant (Philip et al., 2025). Diversity patterns were consistent across technical replicates (Figure 6), confirming reproducibility of PUDU long-read processing under low-diversity conditions.

We note that this community low complexity means this case study is best interpreted as a demonstration of PUDU long-read module function and output structure rather than as an ecological analysis. The important point for PUDU users is that the same workflow, the same configuration structure, and the same PUDUVisR visualization interface operate across this fundamentally different data type with no modification, and that the outputs are immediately available for downstream ecological analysis in the same R-based ecosystem used for the WGS results.

### Minimal working example and reproducibility demonstration

To illustrate the accessibility and reproducibility of PUDU, the complete workflow for each case study can be reproduced from raw reads using three steps: (i) clone the repository, (ii) configure the single YAML file (config.yaml) with sample names, database paths, and module flags, and (iii) execute a single Snakemake command:

git clone https://github.com/amedagliamata/PUDU_pipeline.git

cd PUDU_pipeline

# Edit config.yaml: sample names, database paths, module flags

snakemake --use-conda --cores 8

The complete config. yaml file used is provided in Supplementary Data 1. A step-by-step tutorial covering all three supported sequencing modalities, including annotated configuration examples, output descriptions, and PUDUVisR setup, is provided in Supplementary Data 2.

### Computational performance

PUDU was evaluated on a Linux workstation with 10 CPU threads and 64 GB RAM. Full runtime and peak memory usage per module and dataset are reported in Supplementary Table S1. Peak memory usage remained below 45 GB under the 10-thread configuration, primarily driven by Centrifuger GTDB r226 index (∼43 GB); users with less available RAM should substitute the reduced GTDB index or the Kraken2 standard-8 database (approximately 8 GB). Preprocessing, Bracken re-estimation, and Krona generation are computationally inexpensive (seconds to minutes); Centrifuger is the most memory-intensive step. These resource requirements indicate that PUDU is deployable on mid-range laboratory workstations, a design constraint that directly reflects the access-oriented goals of the project, while also being portable to HPC environments for larger datasets or databases via the Snakemake HPC profile system.

### Limitations of use

Several limitations of PUDU should be acknowledged explicitly. First, PUDU is focused on taxonomic profiling and ecology-oriented reporting and does not implement assembly, metagenome-assembled genome (MAG) recovery, or functional annotation. Users requiring these analyses should use dedicated tools such as ATLAS, SqueezeMeta, or nf-core/mag. Second, taxonomic resolution and classification accuracy remain contingent on reference database completeness; environments that are underrepresented in current databases (e.g., deep-sea, extreme terrestrial, or poorly characterized habitats) may yield higher unclassified fractions. Third, the case studies presented here are based on small sample sizes (n = 6 and n = 3), appropriate for demonstration purposes but insufficient to draw strong statistical conclusions; PUDU outputs are designed to support, not substitute for, well-powered ecological study designs. The current demonstration includes WGS (Kraken2/Centrifuger) and long-read 16S rRNA gene (Emu) modules.

## Discussion

### Comparison with related tools

Table 2 contextualizes PUDU among existing tools for environmental microbiome analysis. QIIME2 is the dominant reference framework for amplicon-based studies and offers extensibility via plugins but is not designed as a unified solution for WGS and long-read profiling within a single configuration and standardized output system. Sunbeam provides a reproducible WGS workflow but does not address amplicon or long-read data. SqueezeMeta offers the most comprehensive WGS functionality, including assembly and MAG recovery, but its resource requirements and complexity place it outside the accessible workstation-deployable range targeted by PUDU. nf-core/ampliseq provides amplicon processing within the Nextflow ecosystem but does not extend to WGS or long-read 16S data. The distinct contribution of PUDU is the combination of multi-modality support, harmonized outputs across classifiers, integrated visualization, and simplified deployment, specifically designed for environmental scientists without dedicated bioinformatics support rather than for expert users seeking maximum analytical power.

**TABLE 2:** Comparative overview of PUDU and related tools for environmental microbiome analysis.

Feature	PUDU	QIIME2	Sunbeam	SqueezeMeta	nf-core/ampliseq
Amplicon 16S rRNA gene	✓	✓			✓
WGS shotgun metagenomics	✓	Limited	✓	✓	
Long-read 16S rRNA gene	✓	Limited			
Assembly and MAG recovery			Limited	✓	
Functional annotation				✓	
Integrated QC (MultiQC)	✓	Limited	✓	✓	✓
Harmonized multi-classifier outputs	✓				
Phyloseq-compatible output	✓	✓			✓
Integrated shiny visualization	✓				
Single-file YAML configuration	✓				
Workstation deployable (≤64 GB RAM)	✓	✓	✓		✓

PUDU is intended for the complementary and common use case of taxonomic community profiling and ecological diversity analysis from raw reads, which represents a substantial proportion of environmental microbiome studies.

### Comparison of visualization tools

Table 3 positions PUDUVisR relative to existing interactive microbiome visualization platforms. Tools such as Animalcules, MetaDAVis, and microbiomeExplorer provide valuable interactive environments but are designed to accept pre-formatted inputs that the user must produce externally. PUDUVisR is distinct in its native coupling to the PUDU output structure: it accepts both raw classifier reports (Kraken2/Bracken, Centrifuger) and Phyloseq objects from the DADA2 module. This native coupling means that the figures and taxa tables exported from PUDUVisR are guaranteed to reflect the pipeline-defined outputs without intermediate manipulation, supporting reproducibility and reducing the risk of inconsistencies introduced by manual data handling.

**TABLE 3:** Comparison of PUDUVisR with existing interactive microbiome visualization tools.

Feature	PUDUVisR	Animalcules	MetaDAVis	microbiomeExplorer
Native pipeline integration	✓			
Accepts Kraken2/Centrifuger reports	✓			
Accepts phyloseq objects	✓	✓		✓
Alpha diversity (shannon, simpson, InvSimpson)	✓	✓	✓	✓
Multiple alpha diversity plot types	✓			
Beta diversity (PCoA, NMDS, PCA)	✓	✓	✓	✓
Taxonomic composition (interactive barplots)	✓	✓	✓	✓
Shared taxa (UpSet plots)	✓			
Metadata-aware filtering and stratification	✓	✓	✓	✓
Export publication-ready figures	✓	✓		✓

### Methodological flexibility and analytical robustness

The multi-classifier design of PUDU enables within-study assessment of analytical robustness that is typically unavailable in single-classifier workflows. Because Kraken2, Centrifuger, and Emu produce outputs in a harmonized format, researchers can directly compare community-level conclusions across tools without reprocessing data or reformatting results. The concordance observed here between Kraken2 and Centrifuger (Spearman ρ = 0.936 at phylum level; PERMANOVA R^2^ = 0.87 and 0.95, respectively) illustrates the practical value of this design as community-level taxonomic patterns were consistent across classifiers despite differences in reference databases and algorithmic approaches. This form of internal cross-validation is particularly relevant in environmental metagenomics, where reference database composition and classification algorithm interact with community structure in ways that are not always predictable, and where differences between classifiers can reflect database coverage gaps rather than noise (Wright et al., 2023).

The modular architecture of PUDU, whereby each analytical step is implemented as an independent Snakemake rule with a dedicated Conda environment, makes the integration of additional classifiers straightforward. Future development will prioritize support for alternative taxonomic methods such as MetaPhlAn and Kaiju, further expanding the pipeline applicability and versatility across diverse metagenomics workflows.

### Scalability and limitations

PUDU is designed to scale from single-user workstations to HPC environments without modification to the workflow code. On HPC systems, Snakemake native cluster execution profiles allow rules to be submitted as independent jobs, enabling parallel processing of large sample cohorts. On workstations, scalability is primarily constrained by memory: users operating below the requirements of the full Centrifuger or Kraken2 databases can substitute reduced-size alternatives with limited impact on community-level resolution. Runtime scales approximately linearly with sample number per module, and Snakemake parallel execution of independent samples further reduces wall-clock time on multi-core systems. Rapid adaptation to new settings is supported by the single-file YAML configuration: database paths, taxonomic rank targets, and preprocessing thresholds can all be modified without altering workflow code, enabling deployment on new datasets with minimal setup effort.

Beyond the environmental contexts shown in this study, PUDU is broadly applicable to a wide range of microbiome research settings, including soil, freshwater, host-associated, and clinical microbiomes, assuming that appropriate reference databases are available (Zhang et al., 2023; Martí et al., 2025). However, environments underrepresented in current reference databases including deep-sea sediments, extreme terrestrial habitats, and poorly characterized host-associated communities will yield higher unclassified fractions, and taxonomic conclusions should be interpreted accordingly. This constraint is not specific to PUDU but is inherent to database composition and taxonomic quality (Edwin et al., 2024; Smith et al., 2022). In this regard, PUDU ensures that unclassified fractions are reported consistently across all classifiers, supporting transparent and comparable assessment of database coverage.

The standardization enabled by frameworks such as PUDU has implications beyond individual studies. Consistent output structures across sequencing modalities and classifiers facilitate cross-study meta-analyses and reproducible community comparisons that would otherwise be hindered by inconsistent processing methods. As the volume of environmental microbiome data continues to grow, the availability of standardized, compatible pipelines becomes increasingly important for enabling synthesis across studies, reducing analytical batch effects, and supporting reproducibility.

PUDU provides an end-to-end, reproducible framework for taxonomic profiling and ecological visualization of environmental microbiomes across amplicon 16S rRNA gene, WGS metagenomics, and long-read full-length 16S rRNA gene data. It is designed for reproducibility and standardization: a single YAML configuration ensures consistent parameterization, Conda-managed dependencies enable portable execution across computing environments, and harmonized output structures across classifiers and modalities enable direct integration with Phyloseq-based ecological workflows and support within-workflow cross-classifier comparisons without additional post-processing. The integrated PUDUVisR visualization layer further reduces post-processing complexity by operating natively on pipeline outputs.

The two case studies presented, Antarctic rhizosphere WGS metagenomics and marine sediment long-read 16S rRNA amplicon sequencing, demonstrate that PUDU recovers ecologically consistent community-level patterns on a mid-range workstation, with harmonized outputs that allow immediate downstream analysis and visualization. The Phyloseq-compatible output structure of PUDU is explicitly designed to enable compositional-aware analyses, including centered log-ratio (CLR) transformation, ANCOM, and ALDEx2, which account for the compositional nature of sequencing count data. Researchers are encouraged to apply such methods where appropriate. We believe that accessible, reproducible, and standardized analytical infrastructure of this kind is a practical prerequisite for equitable participation in global environmental microbiome research, and that PUDU represents a concrete step toward this goal.

## Data Availability

The PUDU pipeline homepage and code can be found at https://github.com/amedagliamata/PUDU_pipeline and PUDUVisR can be found at https://github.com/amedagliamata/PUDUVisR. The datasets used to demonstrate the tool functionality were retrieved from the NCBI Sequence Read Archive (SRA) under BioProject IDs PRJNA419970 and PRJNA1153974.
