# Supplementary material for: PUDU (pipeline for universal diversity unveiling): an accessible end-to-end workflow for taxonomic profiling and ecological visualization of environmental microbiomes across amplicon, shotgun, and long-read sequencing
Source: Front Bioinform. 2026 Aug 11;6:1909327. doi: 10.3389/fbinf.2026.1909327 (PMC13503585; doi:10.3389/fbinf.2026.1909327)
Supplement: Supplementary file 1 [file DataSheet1.pdf]

Pipeline for Universal Diversity Unveiling: Workflow Tutorial and Minimal Working Examples

1. Introduction

This tutorial provides step-by-step instructions for installing, configuring, and executing PUDU across its three supported sequencing modalities: (i) Illumina short-read whole-genome shotgun (WGS) metagenomics, (ii) Illumina short-read amplicon 16S rRNA gene sequencing processed with DADA2, and (iii) Oxford Nanopore long-read full-length 16S rRNA gene sequencing processed with Emu. For each scenario a minimal working configuration is provided that can be adapted directly to new datasets by editing a single YAML file.

PUDU does not require prior experience with workflow management systems. The only prerequisites are basic command-line familiarity and Conda environment management.

2. Prerequisites

The following software must be installed before running PUDU:

- Conda (Miniconda ≥ 4.8 or Anaconda) for dependency management
- Snakemake ≥ 5.18.0 installed into a dedicated Conda environment (see Section 3)
- Git for cloning the repository

All tool-specific dependencies (Trimmomatic, NanoFilt, FastQC, NanoPlot, MultiQC, Kraken2, Bracken, Centrifuger, DADA2, Emu, Krona) are automatically installed by Snakemake into isolated Conda environments on the first run. All reference databases must be downloaded separately and their paths defined in the configuration file.

3. Installation

Clone the PUDU repository and create the Snakemake Conda environment:

```
# 1. Clone the repository
git clone https://github.com/amedagliamata/PUDU_pipeline.git
cd PUDU_pipeline

# 2. Create and activate the Snakemake environment
conda create -n pudu 'snakemake>=7.0.0' -c bioconda -c conda-forge
conda activate pudu
```

The repository contains the following top-level structure:

```
PUDU_pipeline/
├── Snakefile              # Main workflow definition
├── config.yaml            # Central configuration file
├── wrappers/              # Per-tool Conda environment definitions
├── raw_fastq/             # Place input FASTQ files here
└── experiment_design/
    └── metadata.tsv       # Metadata (required for Emu and DADA2)
```

4. Input Data Preparation

4.1 FASTQ files

Place raw FASTQ files in the raw_fastq/ directory. PUDU expects files to follow the naming convention {sample_name}_R1.fastq.gz (and {sample_name}_R2.fastq.gz for paired-end data):

```
# Paired-end Illumina (WGS or amplicon)
raw_fastq/
├── sample1_R1.fastq.gz
├── sample1_R2.fastq.gz
├── sample2_R1.fastq.gz
└── sample2_R2.fastq.gz

# Single-end or Oxford Nanopore long reads
raw_fastq/
├── ont_sample1_R1.fastq.gz
└── ont_sample2_R1.fastq.gz
```

4.2 Metadata file

For modules that produce Phyloseq-compatible outputs (DADA2, Emu), a metadata file must be provided at experiment_design/metadata.tsv:

```
sample_name condition   group
sample1     control     A
sample2     treatment   B
```

5. Tutorial 1: Illumina WGS Paired-End Metagenomics

This scenario applies Kraken2/Bracken and Centrifuger to Illumina paired-end shotgun metagenomics data, as used in Case Study 1 of the main manuscript (Antarctic rhizosphere dataset, BioProject PRJNA419970).

5.1 Configuration

Edit config.yaml with the following settings (all other parameters can remain at defaults):

```
# Sample definition
sample_name: ["sample1", "sample2"]   # list all sample names
analysis_type: "full"     # full analysis: QC + trim + classification

# Sequencing technology
is_paired: true                          # paired-end Illumina
long_reads: false

# Active modules
use_kraken: true
use_centrifuger: true
use_dada2: false
use_emu: false

# Taxonomic output level
organism_taxonomic_level: "G"     # G = genus; options: D P C O F G S
```

```
# Preprocessing (Trimmomatic)
trim_adapters: true
trimmomatic_qc_trim_reads: true
trimmomatic_adapters: "NexteraPE-PE"    # or path to custom adapter
FASTA
trimmomatic_leading: 3
trimmomatic_trailing: 3
trimmomatic_slidingwindow: "4:15"
trimmomatic_minlen: 36

# Database paths (edit to match your system and used database)
kraken2_db: "/path/to/kraken2/db "
centrifuger_db: "/path/to/centrifuger/db "

# Kraken2 parameters
kraken2_conf_interval: 0.2              # classification confidence
threshold
kraken2_rlen: 100                        # read length for Bracken
re-estimation
```

5.2 Execution

```
conda activate pudu

# Standard execution
snakemake --use-conda --cores 8
```

5.3 Expected outputs

Upon successful completion, the following outputs will be produced under results/kraken/ and results/centrifuger/:

Parameter	Description / Value
`{sample}_kraken2_report.txt`	Raw Kraken2 classification report with read counts and taxonomic IDs
`{sample}_kraken2_report_bracken.txt`	Bracken re-estimated abundance table at the selected taxonomic level
`{sample}_krona_graph.html`	Interactive hierarchical taxonomy viewer (open in any web browser)
`{sample}_rarefaction.jpg`	Rarefaction curve for sampling depth assessment
`otu_table_G.csv`	Genus-level OTU abundance matrix across all samples

6. Tutorial 2: Oxford Nanopore Long-Read 16S rRNA Sequencing

This scenario applies Emu to Oxford Nanopore full-length (V3–V9) 16S rRNA amplicon data, as used in Case Study 2 of the main manuscript (marine sediment dataset, BioProject PRJNA1153974).

6.1 Configuration

```
# Sample definition
sample_name: ["ont_sample1", "ont_sample2"]
analysis_type: "full"

# Sequencing technology
is_paired: false                         # Oxford Nanopore: single-end
long_reads: true                         # enable long-read mode

# Active modules
use_kraken: false
use_centrifuger: false
use_dada2: false
use_emu: true                               # Emu for full-length 16S

# Taxonomic output level
organism_taxonomic_level: "G"             # G = genus

# Preprocessing (NanoFilt)
nanofilt_length: 1000              # minimum read length (full-length
16S ~1400 bp)
nanofilt_quality: 10               # minimum mean Phred quality
nanofilt_maxlength: 99999999
nanofilt_headcrop: 0
nanofilt_tailcrop: 0

# Database path
emu_db_path: "/path/to/emu/db"
```

6.2 Execution

```
conda activate pudu
snakemake --use-conda --cores 8
```

6.3 Expected outputs

Outputs will be produced under results/emu/:

Parameter	Description / Value
`{sample}_rel-abundance.tsv`	Per-sample relative abundance table at species level
`emu_phyloseq.rds`	Phyloseq-compatible RDS object for direct import into R/PUDUVisR

7. Tutorial 3: Illumina Amplicon Sequencing with DADA2

This scenario applies DADA2 to Illumina paired-end 16S rRNA amplicon data, producing ASV tables and a Phyloseq object.

7.1 Configuration

```
# Sample definition
sample_name: ["amplicon1", "amplicon2"]
analysis_type: "full"

# Sequencing technology
is_paired: true                    # DADA2 requires paired-end reads
long_reads: false

# Active modules
use_kraken: false
use_centrifuger: false
use_dada2: true
use_emu: false

# Preprocessing
trim_adapters: true
trimmomatic_adapters: "TruSeq3-PE"     # adjust to your library prep

# DADA2 parameters
dada2_train_set: "/path/to/silva/silva_nr99_v138.1_train_set.fa.gz"
dada2_sp_assign:
"/path/to/silva/silva_species_assignment_v138.1.fa.gz"
dada2_for_trunc: 280                 # forward read truncation length
dada2_rev_trunc: 260                 # reverse read truncation length
dada2_multithread: true
```

Note: *Truncation lengths (dada2_for_trunc / dada2_rev_trunc) should be chosen after inspecting quality profiles in the raw MultiQC report at qc_reports/raw_multiqc_report.html. A recommended approach is to set analysis_type: "raw_qc" on the first run to inspect quality, then switch to "full" and set truncation lengths accordingly.*

7.2 Execution

```
# Step 1: QC-only run to inspect quality profiles
# In config.yaml: analysis_type: "raw_qc"
snakemake --use-conda --cores 8

# Inspect qc_reports/raw_multiqc_report.html, then set truncation
lengths

# Step 2: Full run
# In config.yaml: analysis_type: "full"
snakemake --use-conda --cores 8
```

7.3 Expected outputs

Outputs will be produced under results/dada2/:

Parameter	Description / Value
`summary.tsv`	Per-sample read count summary at each DADA2 processing step
`phyloseq.rds`	Phyloseq object containing ASV table, taxonomy table, and sample metadata

8. Downstream Visualization with PUDUVisR

PUDUVisR is a Shiny application included with PUDU that operates directly on pipeline outputs without additional data preparation. It can be installed from GitHub and launched in R:

```
# Install (requires devtools)
install.packages("devtools")
devtools::install_github("amedagliamata/PUDUVisR")

# Launch
library(PUDUVisR)
run_app()
```

PUDUVisR accepts: (i) Kraken2/Bracken report files, (ii) Centrifuger report files, and (iii) Phyloseq RDS objects produced by DADA2 or Emu modules. No reformatting or intermediate scripting is required. Available analytical modules include alpha diversity (Shannon, Simpson, Inverse Simpson), beta diversity (PCoA, NMDS, PCA), taxonomic composition barplots, and shared-taxa UpSet visualizations, all metadata-aware.

9. Troubleshooting

Parameter	Description / Value
`Long reads + paired reads error`	Set is_paired: false for Oxford Nanopore data
`DADA2 requires paired reads`	Set is_paired: true; DADA2 does not support single-end data
`Emu single-end error`	Set long_reads: true when using Emu
`Slow conda env resolution`	Add --conda-frontend mamba to the snakemake command
`Memory exceeded`	Use the GTDB reduced index or switch to Kraken2 standard-8 database

PUDU Repository: `https://github.com/amedagliamata/PUDU_pipeline`

PUDUVisR: `https://github.com/amedagliamata/PUDUVisR`
