# Supplementary material for: PUDU (pipeline for universal diversity unveiling): an accessible end-to-end workflow for taxonomic profiling and ecological visualization of environmental microbiomes across amplicon, shotgun, and long-read sequencing
Source: Front Bioinform. 2026 Aug 11;6:1909327. doi: 10.3389/fbinf.2026.1909327 (PMC13503585; doi:10.3389/fbinf.2026.1909327)
Supplement: Supplementary file 2 [file Table1.docx]

**Supplementary Material**

**Supplementary Data 1.** Configuration and metadata file for PUDU pipeline

**Supplementary Data 2.** Step-by-step tutorial for the PUDU pipeline

**Supplementary Table S1.** Runtime and memory performance across taxonomic classification modules and datasets.

| **Stage** | **Short reads** | | | **Long reads** | | |
| --- | --- | --- | --- | --- | --- | --- |
|  | **Elapsed mean (s)** | **CPU total (s)** | **Max memory (MB)** | **Elapsed mean (s)** | **CPU total (s)** | **Max memory (MB)** |
| Preprocessing | 8.68 | 8.54 | 58.59 | 14.54 | 93.09 | 77.70 |
| Rarefaction plot | 3.59 | 22.33 | 197.43 | 4.49 | 35.28 | 194.81 |
| Kraken2 | 68.99 | 568.32 | 8188.14 | 8.30 | 93.12 | 7729.91 |
| Bracken | 0.45 | 12.54 | 59.44 | 0.29 | 21.20 | 59.55 |
| Centrifuger | 511.46 | 25154.80 | 43036.78 | 40.53 | 1771.27 | 43135.85 |
| Emu abundance estimation | NA | NA | NA | 370.01 | 37738.39 | 4898.64 |
| Krona graph creation | 11.26 | 42.71 | 909.13 | 3.09 | 72.28 | 896.16 |

**Supplementary Table S2.** Parameter configuration used in PUDU pipeline case studies.

| **YAML parameter** | **Parameter description** | **Case Study 1 (WGS, PRJNA419970)** | **Case Study 2 (Long-read 16S, PRJNA1153974)** | **Software default** |
| --- | --- | --- | --- | --- |
| trimmomatic_leading | Remove leading bases with Phred quality below this threshold | 3 | N/A | 3 |
| trimmomatic_trailing | Remove trailing bases with Phred quality below this threshold | 3 | N/A | 3 |
| trimmomatic_slidingwindow | Trim when mean Phred in sliding window drops below threshold (format: window:quality) | 4:15 | N/A | 4:15 |
| trimmomatic_minlen | Discard reads shorter than this length (bp) after trimming | 36 | N/A | 36 |
| nanofilt_length | Minimum read length (bp) to retain; reads below threshold are discarded | N/A | **50** | 0 |
| nanofilt_quality | Minimum mean Phred quality score; reads below threshold are discarded | N/A | **10** | 0 |
| nanofilt_maxlength | Maximum read length (bp) to retain (effectively no upper filter) | N/A | 99999999 | 99999999 |
| nanofilt_headcrop | Number of bases trimmed from the start of each read | N/A | 0 | 0 |
| nanofilt_tailcrop | Number of bases trimmed from the end of each read | N/A | 0 | 0 |
| kraken2_conf_interval | Confidence threshold (0–1); 0 = retain all k-mer matches regardless of confidence | **0** | N/A | 0.2 |
| kraken2_min_base_qual | Minimum base quality for k-mer inclusion | 0 | N/A | 0 |
| kraken2_rlen | Expected read length (bp) used by Bracken for abundance re-estimation | 100 | N/A | 100 |
| kraken2_kmer | K-mer length used in the Kraken2 database build | 35 | N/A | 35 |
| centrifuger_min_hitlen | Minimum alignment length (bp) for a valid classification hit | **18** | N/A | 22 |
| centrifuger_max_hits | Maximum number of distinct alignments reported per read | **3** | N/A | 5 |
